# Candidate Biomarkers to Distinguish Spinal Tuberculosis From Mechanical Back Pain in a Tuberculosis Endemic Setting

**DOI:** 10.3389/fimmu.2021.768040

**Published:** 2021-11-18

**Authors:** Theresa N. Mann, Johan H. Davis, Gerhard Walzl, Caroline G. Beltran, Jacques du Toit, Robert P. Lamberts, Novel N. Chegou

**Affiliations:** ^1^ Division of Orthopaedic Surgery, Department of Surgical Sciences, Faculty of Medicine and Health Sciences, Stellenbosch University, Cape Town, South Africa; ^2^ Institute of Orthopaedics and Rheumatology, Mediclinic Winelands Orthopaedic Hospital, Stellenbosch, South Africa; ^3^ DSI-NRF Centre of Excellence for Biomedical Tuberculosis Research, South African Medical Research Council Centre for Tuberculosis Research, Division of Molecular Biology and Human Genetics, Faculty of Medicine and Health Sciences, Stellenbosch University, Cape Town, South Africa; ^4^ Department of Sport Science, Faculty of Medicine and Health Sciences, Stellenbosch University, Stellenbosch, South Africa

**Keywords:** tuberculosis, spine, biomarkers, back pain, inflammation, cytokines

## Abstract

**Background:**

Spinal tuberculosis (TB) may have a variable, non-specific presentation including back pain with- or without- constitutional symptoms. Further tools are needed to aid early diagnosis of this potentially severe form of TB and immunological biomarkers may show potential in this regard. The aim of this study was to investigate the utility of host serum biomarkers to distinguish spinal TB from mechanical back pain.

**Methods:**

Patients with suspected spinal TB or suspected mechanical back pain were recruited from a tertiary hospital in the Western Cape, South Africa, and provided a blood sample for biomarker analysis. Diagnosis was subsequently confirmed using bacteriological testing, advanced imaging and/or clinical evaluation, as appropriate. The concentrations of 19 host biomarkers were evaluated in serum samples using the Luminex platform. Receiver Operating Characteristic (ROC) curves and General Discriminant Analysis were used to identify biomarkers with the potential to distinguish spinal TB from mechanical back pain.

**Results:**

Twenty-six patients with spinal TB and 17 with mechanical back pain were recruited. Seven out of 19 biomarkers were significantly different between groups, of which Fibrinogen, CRP, IFN-γ and NCAM were the individual markers with the highest discrimination utility (Area Under Curve ROC plot 0.88-0.99). A five-marker biosignature (CRP, NCAM, Ferritin, CXCL8 and GDF-15) correctly classified all study participants after leave-one-out cross-validation.

**Conclusion:**

This study identified host serum biomarkers with the potential to diagnose spinal TB, including a five-marker biosignature. These preliminary findings require validation in larger studies.

## Introduction

Tuberculosis (TB) remains a global health priority with an estimated 10 million people worldwide developing TB disease in 2019 (1). Spinal TB is the most common form of osteoarticular TB and involves a chronic inflammatory process that gradually destroys the bony tissue of one or more vertebrae (2). With progression of the disease, those affected may develop serious sequelae such as spinal deformity, spinal instability and neurological deficit (2). These individuals may require costly corrective surgery and are at risk of significant long-term morbidity (2). Conversely, individuals with less advanced disease may be treated with TB medication alone and show largely favorable outcomes (3). Although spinal TB represents only 1-3% of all TB cases (4–6), the absolute number of individuals affected may be considerable in countries with a high burden of TB (7–9), creating a need for vigilance and effective methods of screening and early diagnosis.

Spinal TB is an insidious disease with a variable and non-specific clinical presentation, factors that contribute to a typical diagnostic delay of 4-12 months or more (6–8, 10–12). The most common symptom is chronic back pain, with 83-100% of individuals presenting with this complaint (7, 8, 11–14). However, previous reports suggest that only approximately 23-56% of patients have constitutional symptoms (e.g. fever, weight loss) (7, 8, 11–14) and 7-23% concomitant pulmonary TB (8, 10, 13, 14). With progression of the spinal TB disease, some patients may develop clinical signs such as kyphotic deformity (7, 8, 11–14). However, for the purposes of early diagnosis, there may not always be obvious features to help distinguish spinal TB amidst a high prevalence of mechanical back pain.

Conventional tools to help screen for pathological back pain at primary care level include evaluation of “red flags” and, when available, blood tests for erythrocyte sedimentation rate (ESR) and/or C-reactive protein (CRP) (15, 16). Red flags are patient factors and clinical signs thought to increase the likelihood of serious disease and include items such as unexplained weight loss (17–19). However, the list of potential red flags is lengthy, may vary with different clinical guidelines and is seldom supported by evidence for the diagnostic accuracy of the items (19). For example, there is a paucity of evidence for the effectiveness of red flags in detecting spinal infections (18). Furthermore, this screening approach may have limited sensitivity with a previous study reporting that the absence of red flags did not decrease the probability of a serious spinal pathology diagnosis (20). When considering ESR and CRP, these markers are elevated in most patients with spinal TB but may remain normal in some cases (12). Furthermore, these are non-specific tests of inflammation and patients may present with elevated levels for a variety of other reasons (15, 16).

Given the limitations of existing screening tools, there could be a significant benefit in more accurate point-of-care triage tests to indicate a high probability of spinal TB or diagnostic tools to identify spinal TB, depending on the performance characteristics of such new tools. One approach that may have potential in this regard is to explore a wider variety of diagnostic blood-based biomarkers than ESR and CRP and, in particular, multi-biomarker signatures. This methodology has received considerable attention in the context of pulmonary TB with certain immune biomarkers showing strong diagnostic potential (21–25). For example, in a recent pan-African study, a two-biomarker signature had a sensitivity of 93% and a specificity of 68% for the diagnosis of pulmonary TB regardless of HIV status (26). The ultimate aim of the afore-mentioned research is to develop cost-effective point-of-care triage or diagnostic tests for pulmonary TB in resource-limited settings (22, 27). However, it is possible that a similar approach could be used to assist with other diagnoses such as spinal TB.

Whereas biomarkers for pulmonary TB have been widely reported (21–26), less is known about biomarker profiles in specific forms of extra-pulmonary TB (EPTB), including spinal TB. Furthermore, the few existing studies describing biomarkers in spinal TB did not explore the diagnostic potential of the biomarkers by comparing them between spinal TB and other back pain etiologies (28, 29). The main aim of the current study was to investigate the utility of host biomarkers to distinguish spinal TB from mechanical back pain. A secondary aim was to test the performance of the afore-mentioned two-biomarker signature for pulmonary TB diagnosis (26) when applied to identify spinal TB among the current participants. It was envisaged that the findings of this exploratory study would identify candidate biomarkers for further evaluation as tools for spinal TB screening and diagnosis.

## Materials and Methods

### Study Design and Setting

This case-control study was conducted at Tygerberg Hospital, a major tertiary hospital in the Western Cape Province of South Africa, between August 2016 and December 2018. In 2019, South Africa had an estimated TB incidence of 615 cases per 100 000 population and was ranked among the top eight countries in world for overall TB burden (1). At least 393 patients with spinal TB were referred to tertiary hospitals in the Western Cape between 2012 and 2015 (9) and, in 2016-2017, spinal TB admissions accounted for 21% of all spine unit admissions to the Tygerberg Hospital (30).

### Participants

Patients with suspected spinal TB were recruited from the orthopedic wards of the hospital according to the following inclusion criteria: ≥ 18 years old, suspected spinal TB based on clinical presentation and Magnetic Resonance Imaging (MRI) findings, and not yet on TB treatment. An exclusion criterion was a subsequent spinal pathology diagnosis other than spinal TB. Patients with suspected mechanical back pain were recruited from the hospital’s spine outpatient clinic according to the inclusion criteria: ≥ 18 years old and ≥ 3 months of chronic back pain of unknown cause. Patients subsequently diagnosed with non-mechanical back pain were excluded.

### Clinical and Demographic Information

Clinical and demographic information for each participant was obtained using a standard intake interview and review of medical records. HIV status and, if applicable, most recent CD4 T-Lymphocyte and HIV viral load levels were obtained from medical records and laboratory test results. Furthermore, the Oswestry Disability Index (31) was completed with each participant in the form of a structured interview in order to provide a measure of disability related to back pain.

### Spine Pathology Diagnosis

Participants with suspected spinal TB underwent an in-theatre spine biopsy for laboratory testing, according to the hospital’s standard procedure. Each participant was subsequently classified as having bacteriologically confirmed- or clinically diagnosed- spinal TB, according to the World Health Organization’s TB reporting definitions (32). Bacteriological confirmation was based on a positive GeneXpert and/or TB culture result from the spine biopsy. Conversely, a clinical diagnosis was made when bacteriological tests were negative for TB but did not present an alternative diagnosis plus factors such as clinical presentation, MRI findings, spine biopsy histology and/or TB bacteriologically confirmed at another site were suggestive of spinal TB.

For participants with suspected mechanical back pain, the diagnosis was based on the evaluation of an orthopedic specialist, including elements such as clinical history, a physical examination, X-rays and, when clinically indicated, advanced imaging.

### QuantiFERON TB Gold Testing

Each participant provided a blood sample for QuantiFERON TB Gold In Tube (QFT) analysis. Blood was collected by venipuncture into QFT tubes (1ml per tube), followed by incubation for 16-24 hours at 37°C in a 5% CO_2_ environment as recommended by the manufacturer (Qiagen, Germany). Thereafter, the tubes were centrifuged at 3000 RCF for 15 min, and supernatants harvested and stored at -80°C until used. Interferon gamma (IFN-γ) responses in the QFT supernatants were measured using the QFT ELISA kit and analyzed and interpreted according to the manufacturer’s software. QFT tests were not standard procedure in the hospital and were conducted for the purpose of the research study.

### Multiplex Host Biomarker Analysis

Blood for investigation of host cytokine levels was collected in 6 ml vacutainer serum tubes (BD Biosciences) and transported to the laboratory at ambient temperatures within 2 hours of collection. Tubes were subsequently centrifuged at 2000 RCF for 10 min and the serum aliquoted into micro-tubes. Aliquots were then stored at -80°C until analysis.

Nineteen host biomarkers were included in the Luminex immunoassay, as listed in **Table 1**. For the most part, these biomarkers were identified from existing TB biomarker literature (22, 27, 33). However, OPG was included due to its role in bone remodeling (34). It was hypothesized that markers of bone metabolism may be of value in differentiating spinal TB given the pathogenesis of this type of TB.

**Table 1:** Host biomarkers included in the Luminex immunoassay.

Abbreviation	Full name	Catalogue number	Detection limit
Biomarkers in kits purchased from R&D Systems Inc., Minneapolis, MN, USA			
CCL1 (I-309)	Chemokine (C-C motif) ligand 1	LXSAHM-11	0.119 pg/ml
CXCL8 (IL-8)	Chemokine (C-X-C motif) ligand 8 (Interleukin 8)	LXSAHM-11	1.8 pg/ml
CXCL9 (MIG)	Chemokine (C-X-C motif) ligand 9 (monokine induced by gamma interferon)	LXSAHM-11	23.8 pg/ml
CXCL10 (IP10)	Chemokine (C-X-C motif) ligand 10 (Interferon gamma-induced protein 10)	LXSAHM-11	1.18 pg/ml
Factor D (Adipsin)	Factor D (Adipsin)	LXSAHM-03	232 pg/ml
Ferritin	Ferritin	LXSAHM-03	1.29 pg/ml
GDF-15	Growth Differentiation Factor 15	LXSAHM-11	1.2 pg/ml
ICAM-1	Intercellular Adhesion Molecule 1	LXSAHM-11	87.9 pg/ml
IFN-γ	Interferon gamma	LXSAHM-11	0.40 pg/ml
IL-10	Interleukin 10	LXSAHM-11	1.6 pg/ml
MPO	Myeloperoxidase	LXSAHM-03	26.2 pg/ml
OPG	Osteoprotegerin	LXSAHM-11	3.62 pg/ml
VEGF-A	Vascular endothelial growth factor A	LXSAHM-11	2.1 pg/ml
VCAM-1	Vascular Cell Adhesion Molecule 1	LXSAHM-11	238 pg/ml
Biomarkers in kits purchased from Merck Millipore, Billerica, MA, USA			
ApoA1	Apolipoprotein A1	HNDG1MAG-36K	0.022 ng/ml
CFH	Complement Factor H	HNDG1MAG-36K	0.037 ng/ml
CRP	C-reactive protein	HCVD3MAG-67K	0.004 ng/ml
Fibrinogen	Fibrinogen	HCVD3MAG-67K	0.004 ng/ml
NCAM	Neural cell adhesion molecule	HNDG3MAG-36K	4.81 pg/ml

The sensitivities of the biomarkers evaluated in reagent kits purchased from R&D Systems are available on the manufacturer’s assay configuration webpage: https://www.rndsystems.com/luminex/analytes. The sensitivities of biomarkers evaluated in kits purchased from Merck Millipore are available in the minimum detectable concentration (MinDC) tables that are included in all of the manufacturer’s kit protocols.

Experiments were conducted in a blinded manner by a Luminex-certified technician, in an ISO15189 accredited laboratory, using the Bio-Plex platform (Bio-Rad Laboratories, Hercules, USA), and were performed according to the kit manufacturer’s instructions. Bio-Plex Manager software version 6.1 was used for bead acquisition and for the analysis of median fluorescence intensities.

### Statistical Analysis

Continuous data are presented as median and interquartile range (IQR) and categorical data as frequency and percentage. Differences in individual biomarker levels between spinal TB and mechanical back pain were investigated using the Mann-Whitney U test for data with non-parametric distribution. The utility of each biomarker to distinguish between groups was assessed using Receiver Operating Characteristic (ROC) curves. Associations between participant characteristics and spinal TB and between participant characteristics and biomarker levels were investigated using a Mann-Whitney U test for continuous data and a Chi-squared or Fisher’s exact test for categorical data. Associations between biomarker levels and spinal TB following adjustment for a participant characteristic were investigated using binomial logistic regression. The utility of combinations of biomarkers to identify spinal TB was investigated using General Discriminant Analysis (GDA) with leave-one-out cross validation. In addition to the GDA model generated from the current data, the performance of an existing pulmonary TB two-biomarker signature, CRP and CCL1 (26), was also evaluated. Finally, an exploratory Principal Component Analysis (PCA) was conducted to investigate linear combinations within the biomarker data. Data was log-transformed or winsorized as necessary prior to logistic regression, GDA and PCA. Analyses were conducted using Graphpad Prism (version 6.00, GraphPad Software, La Jolla, California, USA), jamovi (Version 1.1.9)(https://www.jamovi.org), Statistica (version 14, TIBCO Software Inc.) and the pROC (35) and UBbipl packages in R. Significance was accepted at p < 0.05.

## Results

### Participant Characteristics

Twenty-eight patients with suspected spinal TB and 18 with mechanical back pain were recruited for the study. Two patients with suspected spinal TB were subsequently diagnosed with cancer and excluded. In addition, one patient with mechanical back pain was excluded after advanced imaging was suggestive for infection. The characteristics and clinical presentation of the remaining participants are shown in **Table 2**.

**Table 2:** Participant characteristics and clinical presentation.

		Spinal TB	Mechanical back pain	
		(n = 26)	(n = 17)	
Age, years, median [IQR]		48 [34-56]	53 [46-58]	p = 0.09
Gender, n (%)				p > 0.99
Male		12 (46)	7 (41)	
Female		14 (54)	10 (59)	
HIV status, n (%)				p = 0.02*
Positive		13 (50)	2 (12)	
Negative		11 (42)	12 (71)	
Unknown		2 (8)	3 (18)	
HIV positive, CD4+ T-lymphocytes, n (%)				
< 200 cells/µL		2 (15)	1 (50)	
200 – 499 cells/µL		3 (23)	0 (0)	
≥ 500 cells/µL		3 (23)	0 (0)	
Not available		5 (38)	1 (50)	
HIV positive, Log viral load, n (%)				
< 2.0 log copies/mL		7 (54)	1 (50)	
2.0 – 3.9 log copies/mL		2 (15)	0 (0)	
4.0 – 5.0		2 (15)	1 (50)	
Not available		2 (15)	0 (0)	
TB history, n (%)				p = 0.31
Previous TB		9 (35)	3 (18)	
No previous TB		17 (65)	14 (82)	
QuantiFERON test, n (%)				p = 0.02^†^
Positive		19 (73)	8 (47)	
Negative		2 (8)	7 (41)	
Indeterminate		1 (4)	1 (6)	
Not done		4 (15)	1 (6)	
Back pain duration, months, median [IQR]		6 [3-10]	24 [15-48]	p < 0.01
Constitutional symptoms^‡^, n (%)				p < 0.01
Yes		15 (58)	2 (12)	
No		11 (42)	15 (88)	
Concurrent pulmonary TB, n (%)				p = 0.51
Yes		2 (8)	0 (0)	
No		24 (92)	17 (100)	
Oswestry Disability Index category n (%)				p < 0.01
Minimal or moderate disability		3 (12)	10 (59)	
Severe disability or crippled		10 (38)	5 (29)	
Bed bound		13 (50)	2 (12)	

TB, tuberculosis; IQR, inter-quartile range. Continuous variables were compared using a Mann-Whitney U test and categorical variables were compared using a Chi-squared or Fisher’s exact test. *HIV positive vs. negative or unknown, ^†^QFN positive vs QFN negative, ^‡^Constitutional symptoms include fever, weight loss and malaise.

Thirteen participants with spinal TB and two participants with mechanical back pain were HIV-infected. Fourteen of these individuals were diagnosed with HIV previously and were on anti-retroviral treatment at the time of the study. One participant was diagnosed with HIV in the course of investigation for spinal TB and was not yet on antiretroviral treatment due to the need to first initiate TB treatment. CD4+ T-Lymphocyte and viral load values closest to the time of recruitment, up to a maximum of 7 months, are shown in **Table 2** [median time interval for CD4+ T-lymphocyte test *versus* recruitment = 0 months (IQR, 0 – 0.5 months), median time interval for viral load test *versus* recruitment = 0 months (IQR, 0 – 3.5 months)].

### Spine Pathology Diagnosis

A diagnosis of spinal TB was bacteriologically confirmed in 18 (69%) of the 26 participants in the spinal TB group and clinically diagnosed in the remaining 8 (31%) participants. Clinical diagnoses were supported by a response to TB treatment in five participants whereas two participants passed away and one was lost to follow-up before treatment response could be evaluated. Of the 18 participants with bacteriologically confirmed spinal TB, Quantiferon tests were positive for 14 participants and not done for the remaining four. Of the eight participants with clinically-diagnosed spinal TB, Quantiferon tests were positive for five, negative in two and not done in one participant, respectively.

A diagnosis of mechanical back pain was confirmed by an orthopedic specialist with pathology including degenerative disc disease (n = 10), spondylosis (n = 2), stenosis (n = 2), herniated disc (n = 2) and fracture (n = 1). MRI findings contributed to the diagnosis in 13 of the 17 mechanical back pain participants whereas the remainder were diagnosed based on clinical presentation and X-ray findings alone.

### Utility of Individual Biomarkers for Distinguishing Spinal TB

**Figure 1:**
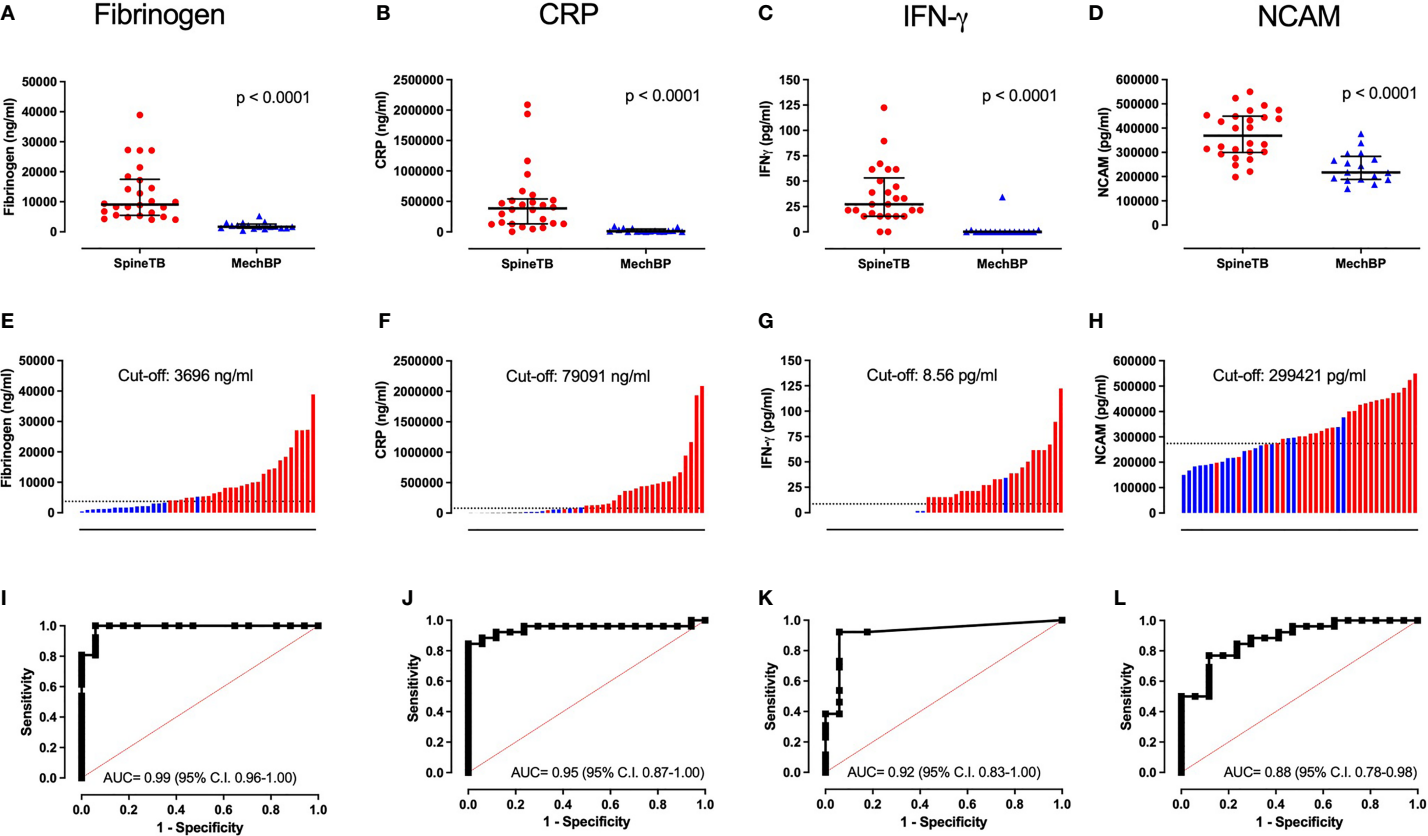
Distribution of individual biomarker levels in spinal TB and mechanical back pain and the utility of biomarkers to distinguish spinal TB. **(A–D)** show the median and interquartile range of biomarkers within each group and the p-value of a Mann-Whitney U test; **(E–H)** show the rank order of individual biomarker levels when combining the groups with red bars representing spinal TB, blue bars representing mechanical back pain and the dashed line representing the cut-off biomarker value for optimal sensitivity and specificity; **(I–L)** show Receiver Operating Characteristic plots along with the Area Under the Curve (AUC) and associated 95% confidence interval. Representative plots for Fibrinogen, CRP, NCAM and IFN-γ are shown, each of which had an AUC ≥ 0.88.

Median biomarker levels in participants with spinal TB and with mechanical back pain are shown in **Table 3**. Of the 19 biomarkers investigated, seven were significantly different between the groups, namely Fibrinogen, CRP, IFN-γ, NCAM, Ferritin, CCL1 and IL-10 (p ≤ 0.04). Each of the afore-mentioned biomarkers was higher among those with spinal TB than among those with mechanical back pain. When evaluating the ROC plot for each biomarker, biomarkers with a significant difference between groups each had an AUC of ≥ 0.68 (**Table 3**). The four individual biomarkers with the highest diagnostic potential were Fibrinogen, CRP, IFN-γ and NCAM (AUC 0.88 – 0.99) (**Figure 1**).

**Table 3:** Biomarker levels and utility of individual biomarkers to distinguish between spinal TB and mechanical back pain.

	Spinal TB	Mechanical back pain	p-value	AUC	AUC	Sensitivity	Specificity
				(95% C.I.)	p-value	(95% C.I.)	(95% C.I.)
Fibrinogen	9120	1688	<0.001	0.99	<0.001	100.0	94.1
	[5729 – 16537]	[1217 – 2155]		(0.96 – 1.00)		(89.1 – 100.0)	(71.3 – 99.9)
CRP	384770	11310	<0.001	0.95	<0.001	88.5	94.1
	[133567 – 517455]	[6176 – 32819]		(0.87 – 1.00)		(69.8 – 97.6)	(71.3 – 99.9)
IFN-γ	27	0.00	<0.001	0.92	<0.001	92.3	94.1
	[16 – 49]	[0.00 – 0.00]		(0.83 – 1.00)		(74.9 – 99.1)	(71.3 – 99.9)
NCAM	368555	217416	<0.001	0.88	<0.001	76.9	88.2
	[301809 – 447342]	[189118 – 271548]		(0.78 – 0.98)		(56.4 – 91.0)	(63.6 – 98.5)
Ferritin	252060	105944	0.002	0.78	<0.001	69.2	82.4
	[164326 – 1.32e+6]	[50653 – 172244]		(0.64 – 0.92)		(48.2 – 85.7)	(56.6 – 96.2)
CCL1	10	6	0.01	0.74	<0.001	73.1	64.7
	[7 – 20]	[5 – 9]		(0.59 – 0.90)		(52.2 – 88.4)	(38.3 – 85.8)
IL-10	0.41	0.00	0.04	0.68	0.02	57.7	76.5
	[0.00 – 1.78]	[0.00 – 0.00]		(0.53 – 0.82)		(36.9 – 76.6)	(50.1 – 93.2)
GDF-15	1054	653	0.12	0.64	0.06	61.5	70.6
	[491 – 1396]	[416 – 853]		(0.48 – 0.81)		(40.6 – 79.8)	(44.0 – 89.7)
CXCL10	50	35	0.15	0.63	0.08	61.5	70.6
	[30 – 61]	[28 – 46]		(0.46 – 0.80)		(40.6 – 79.8)	(44.0 – 89.7)
VEGF-A	192	143	0.15	0.63	0.07	50.0	88.2
	[79 – 359]	[85 – 186]		(0.46 – 0.80)		(29.9 – 70.1)	(63.6 – 98.5)
CXCL9	1933	1575	0.20	0.62	0.10	57.7	88.2
	[1341 – 2949]	[1408 – 1736]		(0.44 – 0.79)		(36.9 – 76.6)	(63.6 – 98.5)
Factor D	3.71e+6	4.17e+6	0.20	0.62	0.10	58.8	65.4
	[3.11e+6 – 4.31e+6]	[3.59e+6 – 4.60e+6]		(0.44 – 0.79)		(32.9 – 81.6)	(44.3 – 82.8)
VCAM-1	1.05e+6	911522	0.22	0.61	0.11	53.8	70.6
	[759996 – 1.46e+6]	[780792 – 1.11e+6]		(0.44 – 0.79)		(33.4 – 73.4)	(44.0 – 89.7)
ApoAI	243608	271259	0.23	0.61	0.11	70.6	53.8
	[218886 – 277761]	[243145 – 308291]		(0.43 – 0.80)		(44.0 – 89.7)	(33.4 – 73.4)
MPO	481671	327798	0.30	0.60	0.15	57.7	70.6
	[254725 – 691086]	[249070 – 502977]		(0.43 – 0.77)		(36.9 – 76.6)	(44.0 – 89.7)
ICAM-1	424590	347445	0.30	0.60	0.15	57.7	70.6
	[307328 – 603615]	[241430 – 451406]		(0.41 – 0.78)		(36.9 – 76.6)	(44.0 – 89.7)
CXCL8	10	17	0.31	0.59	0.16	64.7	61.5
	[6 – 17]	[8 – 23]		(0.41 – 0.78)		(38.3 – 85.8)	(40.6 – 79.8)
OPG	940	1070	0.53	0.56	0.27	53.8	64.7
	[759 – 1258]	[870 – 1295]		(0.38 – 0.74)		(26.6 – 66.6)	(14.2 – 61.7)
CFH	484400	477628	0.89	0.51	0.45	52.9	61.5
	[463443 – 535822]	[460687 – 544841]		(0.33 – 0.70)		(23.0 – 72.2)	(20.2 – 59.4)

TB, tuberculosis; AUC, area under the receiver operator characteristic plot; 95% C.I., 95% confidence interval. Biomarkers are ordered from highest to lowest AUC. Data are expressed as median and interquartile range with between-group differences assessed using a Mann-Whitney U test. CRP, Fibrinogen, ApoA1 and CFH measured in ng/ml and other biomarkers are measured in pg/ml.

### Effect of Demographic and Clinical Characteristics on Individual Biomarkers

When the association between back pain group and participant characteristics was assessed, spinal TB was associated with younger age, HIV infection, shorter duration of back pain symptoms, constitutional symptoms and more severe pain on the Oswestry Disability Index (p < 0.10) (**Table 2**). These demographic and clinical characteristics were also associated with biomarker levels in some cases (**Table 4**). For example, Fibrinogen, CRP, IFN-γ, NCAM and Ferritin were associated with back pain duration (p ≤ 0.03); and Fibrinogen, CRP and IFN-γ were associated with ODI (p ≤ 0.06).

**Table 4:** Association between selected biomarkers and demographic or clinical variables.

	Age		HIV status		QFN result		Back pain duration		Constitutional symptoms		ODI category	
	r^s^	p-value	MW	p-value	MW	p-value	r^s^	p-value	MW	p-value	χ^2^	p-value
Fibrinogen	-0.16	0.32	138	0.30	54	0.01*	-0.55	<0.001*	96	0.002*	10.0	0.04*
CRP	-0.11	0.47	128	0.19	67	0.05	-0.57	<0.001*	116	0.01*	9.10	0.06
IFN-γ	-0.08	0.63	112	0.06	56	0.01*	-0.49	<0.001*	158	0.11	9.99	0.04*
NCAM	-0.28	0.07	139	0.32	60	0.03*	-0.43	0.004*	85	<0.001*	2.92	0.57
Ferritin	0.27	0.09	171	0.98	91	0.27	-0.33	0.03*	179	0.30	4.09	0.39
CCL1	-0.28	0.07	103	0.04*	83	0.17	-0.14	0.37	162	0.15	3.48	0.48
IL-10	-0.21	0.18	125	0.13	93	0.25	-0.19	0.22	200	0.58	1.98	0.74

r^s^, Spearman’s rho; MW, Mann-Whitney U statistic; χ^2^, Kruskal-Wallis statistic. Demographic and clinical variables were selected based on a difference between back pain groups at p < 0.10. The association between biomarkers and these variables was investigated as follows: Spearman’s correlation for continuous variables age and back pain duration; Mann-Whitney U test for binomial variables HIV status, quantiferon (QFN) result and constitutional symptoms; Kruskal-Wallis test for categorical variable Oswestry Disability Index (ODI) category. Only confirmed results were included in the HIV and QFN analyses (n = 15 HIV positive, n = 23 HIV negative) (n = 27 QFN positive, n = 9 QFN negative). *Significant at p < 0.05.

A preliminary investigation of the association between spinal TB and selected biomarkers following adjustment for the afore-mentioned participant characteristics is shown in **Table 5**. Logistic regression yielded very large odd’s ratios in many cases due to large differences in certain biomarkers between back pain groups. Change between the unadjusted and adjusted odd’s ratios suggests that participant characteristics may have influenced the association between back pain etiology and biomarkers. Nevertheless, Fibrinogen, CRP, IFN-γ, NCAM, Ferritin and CCL1 all remained significantly associated with spinal TB following adjustment for age, back pain duration, constitutional symptoms or ODI.

**Table 5:** Association between spinal tuberculosis and selected biomarkers following adjustment for a demographic or clinical variable.

Log_10_ biomarker levels	Unadjusted		Age-adjusted		HIV-adjusted		Back pain duration-adjusted		Constitutional symptoms-adjusted		ODI category-adjusted	
	OR	p-value	OR	p-value	OR	p-value	OR	p-value	OR	p-value	OR	p-value
Fibrinogen	2.3 x10^8^	0.03*	1.5x10^8^	0.04*	3.2 x10^205^	1.00	5.9x10^6^	0.04*	2.8x10^8^	0.03*	6.7x10^7^	0.03*
CRP	35.5	<0.001*	499.2	0.01*	21.2	0.003*	13.8	0.01*	28.8	0.001*	24.8	0.002*
IFN-γ	35.0	<0.001*	36.6	<0.001*	273.5	0.04*	32.8	0.002*	32.0	<0.001*	29.0	<0.001*
NCAM	652891	<0.001*	367153.6	0.001*	405421.5	0.002*	387875.9	0.01*	378429.1	0.003*	9.6x10^6^	0.002*
Ferritin	7.5	0.01*	26.2	0.001*	19.0	0.01*	12.5	0.02*	7.8	0.01*	7.5	0.02*
CCL	13.7	0.02*	11.4	0.04*	7.5	0.08	52.7	0.02*	9.9	0.04*	19.7	0.02*
IL-10	1.6	0.70	1.2	0.87	0.9	0.91	3.2	0.50	2.3	0.52	3.3	0.39

Associations were investigated using binomial logistic regression with mechanical back pain as the dependent variable reference. Only one demographic or clinical variable was included along with the biomarker in each regression analysis. *Significant at p < 0.05

In a limited subgroup analysis, the differences in Fibrinogen, CRP, IFN-γ, NCAM and Ferritin concentrations between the spinal TB and mechanical back pain groups remained significant when including only individuals confirmed as HIV uninfected (spinal TB n = 11, mechanical back pain n = 12) and individuals without constitutional symptoms (spinal TB n = 11, mechanical back pain n = 15) (**Table 6**). Similarly, these differences remained significant when including only individuals ≥ 44 years of age, a cut-off which corresponded to the minimum age in the mechanical back pain group and allowed for a comparable age range between groups (spinal TB, n = 15, median age 54 years, IQR 49-64 years; mechanical back pain, n = 17, median age 53 years, IQR 46-57 years). In contrast, differences in CCL1 and IL-10 were no longer significant in most of these sub-analyses (**Table 6**)

**Table 6:** Biomarker levels and utility of individual biomarkers to distinguish between spinal TB and mechanical back pain in subgroups with no HIV infection, no constitutional symptoms or age 44 years and older.

	HIV uninfected				No constitutional symptoms				Age ≥ 44 years old			
	Spinal TB	Mech BP	p-value	AUC	Spinal TB	Mech BP	p-value	AUC	Spinal TB	Mech BP	p-value	AUC
	(n = 11)	(n = 12)		(95% C.I.)	(n = 11)	(n = 15)		(95% C.I.)	(n = 15)	(n = 17)		(95% C.I.)
Fibrinogen	12808	1688	<0.001	1.00	8172	1688	<0.001	0.98	8882	1688	<0.001	0.99
	[9650 – 17798]	[1184– 2992]		(1.00– 1.00)	[4873 – 2144]	[1217 – 2155]		(0.94 – 1.00)	[7468 – 16483]	[1217 – 2155]		(0.97– 1.00)
CRP	445847	10829	<0.001	0.99	295179	11310	<0.001	0.98	361329	11310	<0.001	1.00
	[168249 – 775863]	[6046 – 22957]		(0.97– 1.00)	[131666 – 469889]	[5655 – 32819]		(0.93 – 1.00)	[142901 – 457868]	[6176 – 32819]		(0.98 – 1.00)
IFN-γ	27.2	0.0	<0.001	0.95	33.0	0.0	<0.001	0.92	33.0	0.0	<0.001	0.93
	[21.3– 33.0]	[0.0 – 0.0]		(0.84– 1.00)	[18.3 – 67.2]	[0.0 – 0.0]		(0.79 – 1.00)	[18.3– 55.9]	[0.0 – 0.0]		(0.83 – 1.00)
NCAM	402549	236484	0.01	0.83	301809	217416	0.01	0.81	312734	217416	0.001	0.82
	[292602 – 446360]	[198490 – 295414]		(0.66– 1.00)	[275984 – 336740]	[187371– 294874]		(0.65 – 0.98)	[273354 – 435458]	[189118 – 271548]		(0.68 – 0.97)
Ferritin	397594	84557	0.002	0.88	460335	70903	0.01	0.79	1.32e+6	105944	<0.001	0.89
	[217407 – 1.32e+6]	[47554 –144536]		(0.73– 1.00)	[174201 – 1315500]	[38257 – 179191]		(0.60 – 0.99)	[254472 – 1.32e+6]	[50653 – 172244]		(0.78 – 1.00)
CCL1	9.3	6.2	0.17	0.67	11.5	6.0	0.04	0.74	8.4	6.0	0.11	0.67
	[6.1– 18.1]	[4.1– 9.2]		(0.45– 0.90)	[6.4 – 19.8]	[2.6 – 8.5]		(0.54 – 0.93)	[6.1– 12.3]	[4.8– 8.5]		(0.48 – 0.86)
IL-10	0.3	0.0	0.21	0.64	0.2	0.0	0.13	0.66	0.0	0.0	0.37	0.58
	[0.0 – 0.9]	[0.0 – 0.1]		(0.40– 0.88)	[0.0 – 2.0]	[0.0 – 0.3]		(0.43 – 0.88)	[0.0 – 0.6]	[0.0 – 0.0]		(0.38 – 0.78)

TB, tuberculosis; Mech BP, Mechanical Back Pain; AUC, area under curve; C.I, confidence interval. Subgroup analyses are presented only for biomarkers that were significantly different between spinal TB and mech BP at the whole-group level. Data are presented as median and interquartile range of biomarker levels with between subgroup differences assessed using a Mann-Whitney U test.

### Utility of Multi-Biomarker Signatures for Distinguishing Spinal TB

**Figure 2:**
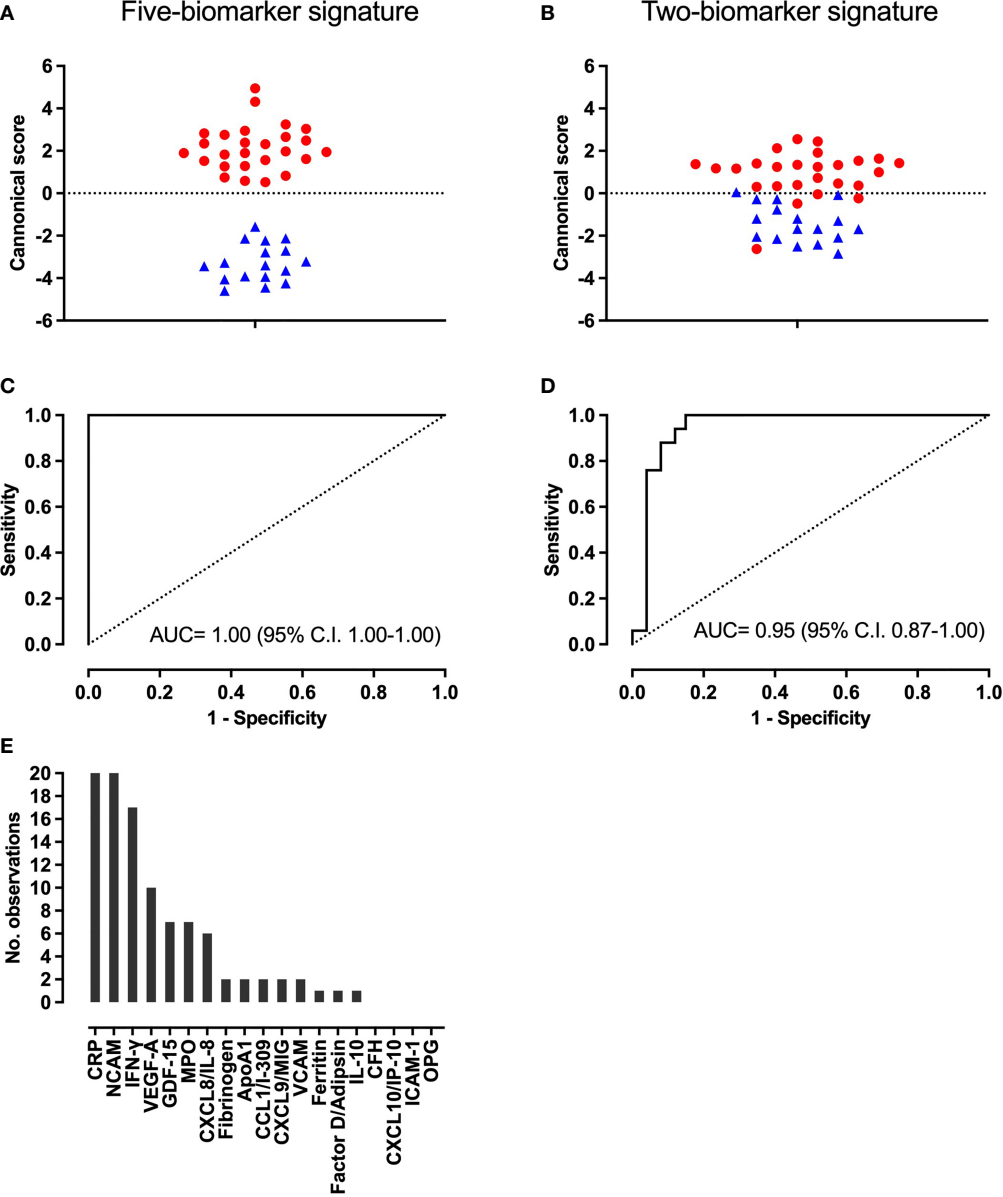
Accuracy of the new five-biomarker signature (CRP, NCAM, Ferritin, CXCL8 and GDF-15) and existing two-marker biosignature (CRP and CCL1) for distinguishing spinal TB and mechanical back pain. **(A, B)** Plot of canonical scores derived from each model, **(C, D)**, Receiver Operating Characteristic plots showing the accuracy of each model and **(E)** Number of times each biomarker appeared in the top 20 five-biomarker General Discriminant Analysis models for discriminating between spinal TB and mechanical back pain. In panels **(A, B)**, red dots and blue triangles represent the scores of individuals with spinal TB and mechanical back pain, respectively.

When evaluating combinations of biomarkers to diagnose spinal TB, GDA modeling identified a five-biomarker signature consisting of CRP, NCAM, Ferritin, CXCL8 and GDF-15 for optimal differentiation of spinal TB *versus* mechanical back pain (**Figure 2**). All biomarkers contributed significantly to the model at p < 0.01. The five-biomarker signature had sensitivity of 100% (95% C.I. 89 – 100%), a specificity of 100% (95% C.I. 84–100%) and a ROC plot AUC of 1.00 (95% C.I. 1.00-1.00). It correctly classified all 43 participants in both re-substitution classification and leave-one-out cross validation analyses. When reviewing the best 20 five-biomarker GDA models, CRP and NCAM appeared in all 20 models, IFN-γ in 17 models and VEGF-A in 10 models. All other biomarkers appeared in ≤ 7 of the 20 best models (**Figure 2**).

For the existing two-biomarker adult pulmonary TB-derived signature, CRP contributed significantly to the model (p < 0.001) but CCL1 did not (p = 0.91). The signature had a sensitivity of 92% (95% C.I. 75–99%), a specificity of 88% (95% C.I. 64–99%) and an AUC of 0.95 (95% C.I. 0.87-1.00)(**Figure 2**). It correctly classified 24 (92%) and 23 (89%) participants with spinal TB in the re-substitution classification and leave-one-out cross validation, respectively.

### Principal Component Analysis

**Figure 3:**
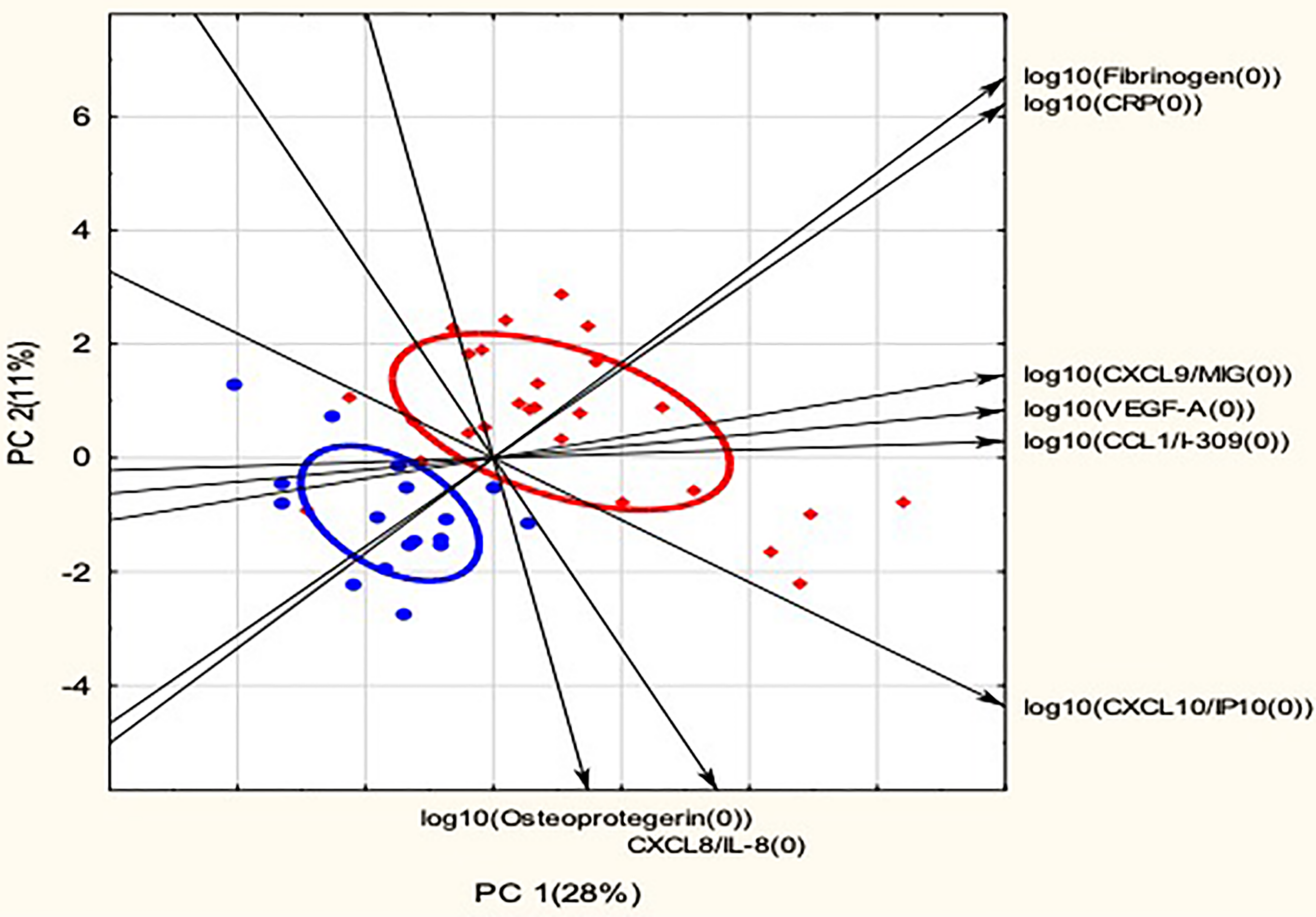
Principal Component Analysis of biomarker distributions in spinal TB and mechanical back pain. Red diamonds indicate scores for spinal TB and blue dots indicate scores for mechanical back pain. Ellipses indicate 50% confidence ellipses for spinal TB and mechanical back pain, respectively. Arrows indicate the direction of increasing biomarker levels. Only biomarkers with R^2^ > 0.40 for principal components 1 and 2 are shown. PC, Principal Component with the percentage of variation explained by the principal component shown in brackets.

To further assess relationships between biomarkers and the two back pain groups, a PCA was performed. A biplot of the first two principal components (PC) identified from the biomarker data is presented in **Figure 3**. PCA generally produced a separation of spinal TB and mechanical back pain scores, although the total variation explained by PC 1 and PC 2 was relatively low at 39%. Fibrinogen and CRP, two of the biomarkers that showed the most potential as individual biomarkers for discriminating between spinal TB and mechanical back pain, were closely correlated and contributed variance to both PC 1 and PC 2. The concentrations of these biomarkers were higher in spinal TB than in mechanical back pain group and they appeared to have the best utility for discriminating between the spinal TB and mechanical back pain groupings. CXCL9, VEGF-A and CCL1 were also closely related and these biomarkers showed a positive association with PC 1. OPG, CXCL8 and CXCL10 showed a negative association with PC 2. PC 1 and PC 2 explained 28% and 11% of variation in the data, respectively.

## Discussion

The current study found that patients with spinal TB had experienced symptoms for several months at the time of diagnosis, in keeping with previous reports (7, 8, 12). Furthermore, the study was one of the few (29) to highlight the functional impact of spinal TB with 88% of patients reporting severe disability as a result of the associated back pain. These observations support the need for interventions that facilitate earlier diagnosis of spinal TB, including the potential role of biomarker-based screening.

When investigating the utility of individual biomarkers, Fibrinogen, CRP, IFN-γ, NCAM and Ferritin showed strong potential to distinguish spinal TB from mechanical back pain (AUC ≥ 0.78). Furthermore these biomarkers generally had similar or slightly improved discriminative utility when restricting the analysis to those without HIV infection, those without constitutional symptoms, or those of a comparable age range. Overall, Fibrinogen and CRP were the most promising individual markers, which is notable in that Fibrinogen is one of the major determinants of ESR (15). ESR and CRP are existing screening measures for a potential infective etiology in back pain with levels typically elevated in spinal TB and normal in chronic mechanical back pain (36). ESR was not assessed in the current study and it is unclear whether Fibrinogen has higher discriminative potential than this conventional measure. Nevertheless, the study demonstrated that conventional marker CRP remained one of the most useful individual markers for distinguishing spinal TB from mechanical back pain despite the range of novel biomarkers investigated.

Although Fibrinogen and CRP performed well when differentiating between the current participants, it is likely that these biomarkers would have lower specificity for spinal TB within a primary care context. Furthermore, they may have lower levels earlier in the disease process or remain normal in a proportion of patients with spinal TB (12). Thus, use of a multi-biomarker signature would be expected to improve diagnostic accuracy and help to inform clinical decision-making among a more heterogeneous patient population. The current study identified a five-biomarker signature (CRP, NCAM, Ferritin, CXCL8 and GDF-15) which was able to correctly classify all participants regardless of HIV status. Nevertheless, the ability of this biomarker signature to identify spinal TB requires further validation, including prospective studies in primary care settings, with inclusion of any patient with chronic back pain, regardless of subsequently elucidated etiologies. Future studies should also consider further investigation of biomarkers that were not included in the best-performing biomarker signature, yet appeared in many of the top 20 GDA models. Examples of this include IFN-γ and VEGF-A, which appeared in 17 and 10 of the best 20 models, respectively. PCA explained a relatively low amount of variation in biomarker levels in the current study, suggesting limited potential for linear combinations of biomarkers in this context. Nevertheless, principal component findings supported further investigation of several of the biomarkers identified through other analyses, including Fibrinogen, CRP, VEGF-A and CCL1.

Validation of the current findings in larger studies is particularly important in light of differences in HIV infection, back pain duration and back pain severity between the spinal TB and mechanical back pain groups. Most of the top-performing biomarkers remained significantly associated with spinal TB following adjustment for participant characteristics. However, the sample size and distribution of the data were notable limitations in these analyses. For example, although biomarker associations remained significant in the subgroup without HIV infection, the study was not able to adequately explore the influence of HIV infection on biomarker utility as only two individuals in the mechanical back pain group were HIV infected. Spinal TB is a comparatively uncommon form of TB and it was necessary to conduct the study at a tertiary hospital in order to optimize recruitment. However, limited resources for this exploratory research meant that it was necessary to recruit participants with mechanical back pain from the same setting. As observed in the study, patients with spinal TB may have developed very severe pain by the time of referral to a tertiary hospital and patients with mechanical back pain may have had pain for a long duration before being referred to a tertiary hospital. Overlap in patient characteristics between the different back pain etiologies would be expected to be larger at primary care level, where biomarker-based screening would provide most benefit.

Previous studies suggest differential cytokine responses in different types of TB (37) and the optimal diagnostic biomarker signatures may likewise vary according to type of TB disease (33). Nevertheless, a test with utility across different types of TB would have practical advantages and it is interesting to compare findings when the inclusion of the same biomarkers allows (33). Two previous studies in pulmonary TB included several of the same biomarkers as in the current study and also used the same analysis platform (22, 27). As with the current study, CRP (22, 27), NCAM (27), IFN-γ (22), Ferritin (27) and Fibrinogen (22) were among the top-performing individual biomarkers in one or both of these previous studies, depending on the other biomarkers included. However, whereas both Fibrinogen and CRP had high diagnostic utility in the current study, Fibrinogen appeared to have lower diagnostic utility in pulmonary TB with one study reporting an AUC of 0.70 for Fibrinogen *versus* 0.86 for CRP (22). Conversely, it was noted that CXCL10 appeared to have higher diagnostic utility in pulmonary TB than in the current study. CXCL10 has received considerable attention as a diagnostic marker for pulmonary TB with an AUC of 0.78 and 0.83 in the afore-mentioned studies and an overall AUC of 0.93 in a recent meta-analysis (38). However, in the current study, CXCL10 was not significantly different between the spinal TB and mechanical back pain groups and had an AUC of only 0.63. Similarly, CXCL10 levels showed poor utility to discriminate between back pain etiology groupings in the PCA. Variation in biomarker utility between different types of TB may be related to factors such as tissue-specific variation in immunopathogenesis and compartmentalization of immune cells (39).

When considering combinations of biomarkers from previous studies, the existing pulmonary TB biosignature of CRP and CCL1 performed well in the current study, achieving an AUC of 0.95 compared to 0.90 in the original test set of suspected pulmonary TB (26). While this is somewhat encouraging, it was noted that CCL1 did not contribute significantly to the model in the current study and the model AUC was the same as that of CRP alone. In the current study, CCL1 was no longer significantly different between spinal TB and mechanical back pain in subgroups without HIV infection or of similar age range and it is possible that this analyte has somewhat less utility for distinguishing spinal TB than pulmonary TB. While this observation requires investigation in larger studies, it makes the point that testing existing biomarker signatures alongside optimal combinations can provide helpful additional insights (40)

The current study had several limitations including the small sample size and the case-control study design. As previously discussed, the study was also limited by differences in certain participant characteristics, such as HIV infection, and was not able to adequately explore the effect of these characteristics on biomarker utility. Due to the exploratory nature of the study, we chose not to correct for multiple testing. The rationale was that such corrections may risk relevant biomarker associations being missed and not carried forward into future studies. Although many of the current biomarker findings are in keeping with previous literature, there remains a risk of false positive results and this constitutes a further limitation of our study.

Although the findings are preliminary, biomarkers that showed potential, either individually or through inclusion in the biomarker models, could be considered candidates for further investigation in larger studies. Given that back pain originating from spinal TB may not always be accompanied by constitutional symptoms, such future studies could consider recruiting all individuals presenting with more than 6 weeks of back pain in a TB endemic setting. These individuals could then be monitored prospectively to detect those diagnosed with spinal TB and those confirmed with an alternative diagnosis. Such studies should also assess the influence of HIV status, including the severity of HIV/AIDS disease, on the diagnostic accuracy of the biomarkers.

In conclusion, the current case-control study evaluated a novel selection of biomarkers for distinguishing spinal TB from mechanical back pain and identified both individual biomarkers and a five-biomarker signature as candidates in this regard. These preliminary findings require validation in larger studies, including prospective cohort studies to assess the accuracy of candidate biomarkers among patients presenting to primary care health facilities with chronic back pain.

## Data Availability Statement

The raw data supporting the conclusions of this article will be made available by the authors, without undue reservation.

## Ethics Statement

This study involving human participants was reviewed and approved by the Health Research Ethics Committee of Stellenbosch University and by hospital management. The patients provided written informed consent to participate in the study.

## Author Contributions

TM: Conceptualization, investigation, formal analysis, project administration, visualization, and writing—original draft. JD: Methodology, investigation, and writing—review and editing. GW: Methodology, resources, and writing—review and editing. CB: Investigation and writing—review and editing. JT: Resources, supervision, and writing—review and editing. RL: Supervision and writing—review and editing. NC: Methodology, investigation, resources, and writing—review and editing. All authors contributed to the article and approved the submitted version.

## Funding

The work was supported by the South African Government through the National Research Foundation (NRF), the South African Research Chairs Initiative (SARChi) in TB Biomarkers (Grant number 86535), the Stellenbosch Spinal Surgery Training Trust, the Stellenbosch University Division of Orthopaedic Surgery Research Fund and the South African Orthopaedic Association (SAOA). However, these parties were not involved in the study design; in the collection, analysis and interpretation of the data; in the writing of the report; or in the decision to submit the article for publication. Furthermore, the views expressed are those of the authors and are not necessarily to be attributed to the funders.

## Conflict of Interest

The authors declare that the research was conducted in the absence of any commercial or financial relationships that could be construed as a potential conflict of interest.

## Publisher’s Note

All claims expressed in this article are solely those of the authors and do not necessarily represent those of their affiliated organizations, or those of the publisher, the editors and the reviewers. Any product that may be evaluated in this article, or claim that may be made by its manufacturer, is not guaranteed or endorsed by the publisher.
